# A New Voice for the Poor

**DOI:** 10.1371/journal.pntd.0000077

**Published:** 2007-10-31

**Authors:** Peter Hotez

**Affiliations:** Sabin Vaccine Institute and Department of Microbiology, Immunology, and Tropical Medicine, George Washington University Medical Center, Washington, D. C., United States of America

The neglected tropical diseases (NTDs) are a group of tropical infections, such as river blindness, hookworm, lymphatic filariasis, leishmaniasis, and trachoma, which represent some of humankind's most ancient scourges and possibly our greatest global health disparities. Largely confined to the unseen rural areas of the developing world, the NTDs were for centuries the forgotten diseases of forgotten people [1]. They are chronic, disabling, and often stigmatizing conditions, and their poverty-promoting features provide a genuine reason why parts of Africa, Asia, and the tropical regions of the Americas cannot escape their low-income status.

Although these diseases have been overshadowed by better-known conditions, especially the “big three”—HIV/AIDS, malaria, and tuberculosis—evidence collected in the past few years has revealed some astonishing facts about the NTDs. They are among the most common infections of the poor—an estimated 1.1 billion of the world's 2.7 billion people living on less than US$2 per day are infected with one or more NTDs. When we combine the global disease burden of the most prevalent NTDs, the disability they cause rivals that of any of the big three. Moreover, the NTDs exert an equally important adverse impact on child development and education, worker productivity, and ultimately economic development. Chronic hookworm infection in childhood dramatically reduces future wage-earning capacity, and lymphatic filariasis erodes a significant component of India's gross national product. The NTDs may also exacerbate and promote susceptibility to HIV/AIDS and malaria.

All hope is not lost. Through the provision of a package of preventive chemotherapy, we have a unique opportunity to reduce the health and economic costs of the NTDs, a strategy that cannot be applied to most other disease entities in the developing world. Such a package may also help to tackle the big three diseases. These drug packages are being administered by a group of NTD partnerships that are working together through the Global Network for NTD Control (http://gnntdc.sabin.org/), while new product development partnerships have been established that will someday produce a revolutionary generation of additional drugs, diagnostics, and vaccines. Taking on the NTDs represents one of the most efficient and cost-effective means of achieving the Millennium Development Goals in the areas of child and maternal health, infectious diseases, poverty reduction, and building global partnerships, and is a step forward in achieving international human rights.

*PLoS Neglected Tropical Diseases* is an important new ally for global efforts to control and eliminate the world's most burdensome NTDs. We believe that our new journal, dedicated to publishing research that can help reduce disease and despair, is a modest yet important contribution to relieving suffering and building research and disease control capacity in developing countries. With unprecedented representation from women and developing countries on our Editorial Board, together with a magazine section devoted to policy, analysis, and debate, we hope that *PLoS Neglected Tropical Diseases* will promote a collective voice for the world's poorest people. And by adopting an open-access model—all articles are freely available worldwide and can be copied, distributed, translated, and built upon provided authors are credited and the source is cited—we hope to reach an audience of 6 billion.

*PLoS Neglected Tropical Diseases* was not launched in a vacuum. From its very beginning, the Public Library of Science's founders—Harold Varmus, Pat Brown, and Mike Eisen—grasped the power of open access for the developing world. Subsequently, the Bill and Melinda Gates Foundation recognized the potential of *PLoS Neglected Tropical Diseases* for building research and public health capacity in developing countries and awarded the journal a grant to cover its launch phase. The announcement of the journal was greeted with tremendous support by the popular press and by other scholarly journals. *The Lancet,* for example, in an editorial praising our launch, wrote: “We welcome this initiative. Any investment in scaling up communication about global health is to be warmly applauded” [2]. With all of this support comes a responsibility. We aspire to make *PLoS Neglected Tropical Diseases* an international resource, one that truly benefits the scientific, medical, and public health communities. This means that we need our readers to tell us how we can be helpful. We exist solely for the benefit of our constituents: the thousands of dedicated scientists, health care professionals, and public health experts, and the poor communities worldwide that you serve every day. This is a journal “run by and for the community,” and we look forward to receiving your best work.
